# Germline *MUTYH* gene mutations are not frequently found in unselected patients with papillary breast carcinoma

**DOI:** 10.1186/1897-4287-12-21

**Published:** 2014-12-12

**Authors:** Ewout P Boesaard, Ingrid P Vogelaar, Peter Bult, Carla AP Wauters, J Han JM van Krieken, Marjolijn JL Ligtenberg, Rachel S van der Post, Nicoline Hoogerbrugge

**Affiliations:** Department of Human Genetics, Radboud university medical center, P.O. Box 9101, 6500 HB Nijmegen, The Netherlands; Department of Pathology, Radboud university medical center, Nijmegen, The Netherlands; Department of Pathology, Canisius Wilhelmina Hospital, Nijmegen, The Netherlands

**Keywords:** *MUTYH*, Papillary carcinomas of the breast, *MUTYH*-associated polyposis

## Abstract

*MUTYH-*associated polyposis (MAP) is an autosomal recessive disease, which predisposes to polyposis and colorectal cancer. There is a trend towards an increased risk of breast cancer in MAP patients, with a remarkable proportion of papillary breast cancers. To determine whether *MUTYH* mutations are associated with this specific and rare type of breast cancer, 53 unselected patients with papillary breast cancer were analyzed for founder mutations in the *MUTYH* gene. No germline mutations were identified, indicating that biallelic *MUTYH* mutations are not a frequent underlying cause for the development of papillary carcinomas of the breast.

## Introduction

*MUTYH-*associated polyposis (MAP) is an autosomal recessive disease, which was first identified by El-Tassan *et al*. in 2002 [1]. Several studies confirmed the association between germline *MUTYH* mutations and colorectal adenomas and colorectal carcinomas [2–4]. The *MUTYH* gene is involved in base excision repair (BER) preventing G:C → T:A conversions. These conversions are caused by oxidative damage forming 8-oxo-7,8-dihydro-2-deoxyguanosine (8-oxoG) resulting in a mis-incorporation of adenine that, if not repaired by *MUTYH* DNA glycosylase, could lead to a G:C → T:A conversion [5–7]. In the Dutch population the mutations p.Y179C, p.G396D and p.P405L account for approximately 90% of all *MUTYH* germline mutations [8, 9].

Several studies on extracolonic cancers in MAP patients have been performed. In one study in MAP patients in which the histological subtype of the breast tumors were included, two out of eleven breast cancers were described as intracystic papillary type [10]. Also, the occurrence of *MUTYH* mutations in patients with breast cancer has been examined. Some of these studies found a significant increase in *MUTYH* mutations in breast cancer patients, while others could not find this potential association. [11–15]. Two of these studies specified the breast cancer histological subtypes of the patients. Out *et al*. found that minor allele genotypes of several *MUTYH* variants showed a trend towards association with lobular BC histology [14], while Rennert *et al*. found no differences for ductal or other BC histology type among 30 patients heterozygous for p.Gly396Asp or p.Tyr179Cys and 359 patients homozygous wild-type for these mutations [15].

Intracystic papillary breast cancer accounts for less than 1% of the total amount of breast cancer [16, 17], however, this type has been observed in an unexpectedly high proportion of MAP patients. We therefore wondered whether papillary carcinomas of the breast could be used as a hallmark to recognize patients with MAP. To explore whether biallelic *MUTYH* mutations are a frequent cause underlying the development of papillary carcinomas of the breast, we analyzed the prevalence of germline *MUTYH* mutations in an unselected patient group with papillary breast cancer.

## Methods

### Patient samples

The study group consisted of 74 patients with papillary carcinomas of the breast, including papillary ductal carcinoma in situ (DCIS), intraductal/intracystic papillary carcinomas and invasive papillary carcinomas, which were diagnosed between January 1990 and December 2012 in the Radboud university medical center, and between January 2002 and December 2012 in the Canisius Wilhelmina Hospital, Nijmegen. Cases were retrieved from the surgical pathology files. Information concerning other malignancies or polyposis coli was obtained from pathology records using the PALGA (nationwide histopathology and cytopathology data archive of the Netherlands) database. All records were anonymously listed in a database. Cases were treated according to the FEDERA (Dutch Federation of Biomedical Societies) code, with anonymous use of redundant tissue for research purposes.

### DNA analysis

DNA was isolated from formalin-fixed paraffin-embedded (FFPE) tissue. Preferably normal (“benign”) tissue was selected. If no benign tissue was available, which was the case for two patients, tissue containing tumor material was used. FFPE tissue was reviewed to exclude presence of tumor cells before and after DNA isolation with H&E-stained slides. DNA concentration of the samples was measured using the Nanodrop ND-1000 (Thermo Fisher Scientific, USA) and Qubit 1.0 (Life Technologies Corporation, UK). The quality of samples was evaluated by performing a DNA size ladder polymerase chain reaction (PCR). Patients for whom the isolated DNA was of insufficient quality were excluded from the study. *MUTYH* primers were developed using the UCSC Browser (Table 1) and the DNA was amplified using PCR. The PCR product was enzymatically purified, after which sequencing was performed using Big-Dye terminator sequencing (BigDye Terminators v 1.1 (Applied Biosystems, USA)). The sequence data was analyzed using Vector NTI advance V11.0 (Invitrogen corporations, USA) and Chromas Lite (Technelysium, Australia). The following three founder mutations were evaluated; c.536A>G (p.Y179C), c.1187G>A (p.G396D) and c.1214C>T (p.P405L). *MUTYH* mutations were annotated according to the most recent version of the reference sequence, NM_001128425.1.

**Table 1:** **Primers used for amplification**

Variant	Forward primer (5' - 3')	Reverse primer (5' - 3')	Product
p.Y179C	CCCCCTAGCTCCTCTACCAC	CGGGTGATCTCTTTGACCTC	202 bp
p.G396D and p.P405L	AGCCCAACGCTGTAGTTCCT	GAGGGCAGTGGCATGAGTAA	182 bp

Bp = Base pair.

## Results

Tissue was available from 72 of in total 74 patients with papillary breast cancer; benign tissue was available from 70 patients and for two patients we used malignant tissue. Patient characteristics are depicted in Table 2. Six patients (8.3%) had a DCIS of the papillary type, 49 patients (68.1%) had an intraductal or intracystic papillary carcinoma with no signs of invasion in the surrounding tissue and in 17 patients (23.6%) an invasive component was found which originated from the intraductal or intracystic papillary carcinoma or papillary type DCIS. Within our total cohort of breast cancer patients, 9 patients were also diagnosed with at least 1 adenoma of the colon and 4 patients with a metachronous or synchronous colon carcinoma were present.

**Table 2:** **Patient characteristics**

Patients, n	Sequenced (N = 53)	All patients (N = 72)
Sex (Male/Female)	52 female, 1 male	71 female, 1 male
Age at diagnosis (range)	67.5 years (range 22–89 years)	67.4 years (range 22–96 years)
Histological type		
– Papillary DCIS	3 (5.7%)	6 (8.3%)
– Intraductal/intracystic PC	39 (73.6%)	49 (68.1%)
– PC with invasive component	11 (20.8%)	17 (23.6%)
Patients with colon adenomas	9 (17.0%)	9 (12.5%)
Patients with colon carcinoma	3 (5.7%)	4 (5.6%)

PC = Papillary carcinoma; DCIS = Ductal carcinoma in situ.

From 53 patients (73.6%), including 9 patients with a colorectal adenoma and 3 patients with a colon carcinoma, the quality of the isolated DNA was sufficient for further use in analysis of the three most common founder mutations in the Dutch population. The average age was 67 years (SD 13 years) with a range of 22 to 96 years, which is comparable with a population wide study on patients with a papillary carcinoma of the breast [18]. No *MUTYH* mutations were found (Table 3).

**Table 3:** **Mutation analysis**

Patients, n	p.Y179C	p.G396D	p.P405L
N = 53	0/53	0/53	0/53

## Discussion

Our data do not confirm that biallelic germline *MUTYH* mutations are a frequent underlying cause of papillary carcinomas of the breast. Such relation was previously suggested by the results of Vogt *et al*. that showed an overrepresentation of intraductal papillary carcinomas of the breast in a MAP cohort [10]. Papillary carcinomas of the breast are a group of tumors with a favorable prognosis [19]. The terminology of papillary carcinomas has been widely disputed. The intraductal and intracystic papillary carcinomas were first considered as a form of carcinoma in situ, but it was shown that they often completely lack a myoepithelial layer and can be invasive [20]. Some authors prefer to use the term encapsulated papillary carcinoma, which refers to intracystic and solid papillary tumors, which are circumscribed and mostly have a fibrous capsule [21]. For this article, we have made a differentiation between papillary DCIS, intraductal or intracystic papillary carcinomas and papillary carcinomas with an invasive component. As the definition of the various histological variants of this malignant tumor differs in the literature, we decided to include in our study all the previously described papillary tumors. In our study, papillomas of the breast were excluded because a relation between this benign tumor and malignant papillary carcinomas has not been described.

In our study we could not identify the association between *MUTYH* mutations and papillary carcinomas of the breast in an unselected group of patients with papillary carcinomas. The current approach has been used in other studies in cancer predisposition syndromes. For example, patients with Familial Adenomatous Polyposis, caused by mutations in the *APC* gene, have a higher risk of developing desmoid tumors, but when a unselected cohort of this rare type of tumor is tested for *APC* mutations, only a small mutation detection rate has been found [22, 23]. The same accounts for patients with endometrial cancer that are screened for Lynch syndrome-predisposing genes [24, 25].

Based on the data presented in this study, an enrichment of biallelic *MUTYH* mutations in other patient cohorts cannot be excluded. This might be true for patients with other histological subtypes of BC, patients with very early onset of papillary carcinoma, patients with papillary carcinomas of the breast together with polyposis as well as patients with a family history of colon carcinomas. Our data, however, do indicate that papillary carcinomas of the breast cannot be used as a hallmark to recognize patients with *MUTYH*-associated polyposis.
